# Validity and Inter-Device Reliability of the OTBeat Burn^TM^ Monitor to Estimate Heart Rate During Exercise

**DOI:** 10.3390/sports13020049

**Published:** 2025-02-08

**Authors:** Matteo F. de Leon, Clayton L. Camic, Matthew J. Herring, Christopher M. Hill

**Affiliations:** 1 Department of Kinesiology and Physical Education, Northern Illinois University, DeKalb, IL 60115, USA; z1927462@students.niu.edu (M.F.d.L.); mherring1@niu.edu (M.J.H.); 2 School of Kinesiology, Louisiana State University, Baton Rouge, LA 70803, USA; chrishill@lsu.edu

**Keywords:** fitness wearables, accuracy, arm-based, cycle ergometry

## Abstract

The purpose of this study was to determine the accuracy and inter-device reliability of the OTBeat Burn^TM^ heart rate monitor during an incremental test to exhaustion on a cycle ergometer. Twenty males (mean ± SD age = 21.1 ± 1.9 years) volunteered to complete a test to exhaustion on a cycle ergometer with OTBeat Burn^TM^ devices placed on the forearm and upper arm, with a 12-lead electrocardiogram used as the criterion. The heart rate was recorded every 30 s and averaged across each two-minute stage. Accuracy was assessed through calculation of the mean absolute percent error (MAPE), Bland–Altman plot, and Lin’s concordance correlation coefficient (CCC). An intraclass correlation coefficient (ICC) was used to assess the inter-device reliability. Statistical significance was set at α < 0.05. The MAPE (±SD), Bland–Altman regression analyses, and Lin’s CCC values were 0.9 (±0.6)% and 0.8 (±0.5)%, r = 0.107 and 0.303, and r*_c_* = 0.998 and 0.998 for the forearm and the upper arm monitors, respectively. The ICC for inter-device reliability was *R* = 0.999. Our findings indicated the OTBeat Burn^TM^ monitors placed on the forearm and upper arm provided highly accurate and reliable values when compared to an electrocardiogram from low to maximal exercise intensities.

## 1. Introduction

Commercially available fitness monitors have become an incredibly popular form of wearable technology for tracking health and performance at rest and during exercise [1,2,3,4]. In fact, the American College of Sports Medicine has named “wearable technology” as the top fitness trend for seven of the last nine years [1], and the global industry of these products is currently valued at approximately 100 billion USD [4], with an estimated 100 million wearables sold annually [5,6]. These wearable devices are used to estimate numerous cardiovascular (heart rate, heart rate variability, percent oxygen saturation, and irregular rhythms); pulmonary (breathing rate); metabolic (oxygen uptake, energy expenditure, lactate threshold, and body temperature); performance (step count, distance covered, duration of exercise, training volume, pace, and acceleration); recovery; sleep; and stress variables [1]. Of these features, the monitoring of the heart rate is often highly beneficial due to its utility in prescribing optimal exercise intensity zones specifically designed for cardiorespiratory fitness, weight loss, and rehabilitation strategies in athletic and clinical populations [1,7,8,9,10]. Previous studies, however, have established that the validity of measuring the heart rate in these devices can be influenced by the mode and intensity of exercise, location of placement on the body, sensor technology, and other factors [6,11,12,13]. Moreover, wide-ranging accuracy values have been reported, with some devices exhibiting measurement errors exceeding 20% or up to 50 beats per minute compared to criterion methods (i.e., electrocardiogram, ECG) [6,8,12,14,15,16,17]. Concerns over these issues continue to be reported [3,4,12], and many authors have proposed more research should be completed on the accuracy, as well as reliability, of these devices [1,16,17,18,19]. Thus, the importance of determining the validity metrics of specific heart rate monitors is highly necessary based on their global popularity, diverse capabilities, practical applications, and overall cost.

Orangetheory^®^ Fitness is a commercial group fitness franchise with over 1500 locations and one million clients worldwide [20]. Their studios offer high-intensity workout classes based on heart rate responses to ensure patrons are within the ideal exercise intensity zones to achieve their individual goals. The OTBeat Burn^TM^ heart rate monitor is the device used by Orangetheory^®^ Fitness, which is recommended to be worn on the forearm or upper arm [21]. This device estimates the heart rate through a noninvasive technique called photoplethysmography (PPG) that utilizes LED optical sensors to detect changes in the blood volume through the emission, absorption, and reflection of infrared light in the underlying vessels [7,22]. Despite being a simple and cost-effective technique found in many heart rate monitors on the market, PPG has shown limitations in measurements at higher exercise intensities that may be due to motion artifact or device location on the body [6,14,15,23]. These limitations and other validity metrics have been extensively examined in many of the most popular brands (e.g., Apple, Garmin, and FitBit), whereas there is a paucity of data concerning lesser known but widely used devices such as the OTBeat Burn^TM^. To our knowledge, the OTBeat Burn^TM^ monitor has yet to be validated across a heart rate range consistent with high-intensity exercise. In addition, the inter-device reliability for this monitor has not been established in the different placement locations (i.e., forearm vs. upper arm). Therefore, the purpose of this study was to determine the accuracy and inter-device reliability of the OTBeat Burn^TM^ heart rate monitor during an incremental test to exhaustion on a cycle ergometer.

## 2. Materials and Methods

### 2.1. Subjects

Twenty college-aged males (mean ± SD age = 21.1 ± 1.9 years; body mass = 84.7 ± 15.0 kg; height = 181.2 ± 7.5 cm) who participated in recreational training (aerobic training = 1.8 ± 1.9 h·wk^−1^; resistance training = 6.2 ± 2.7 h·wk^−1^) volunteered for a single laboratory visit. Inclusion criteria included ages of 19–29 years and not reporting any of the following that could influence the outcome of the study: history of cardiovascular disease; chest pain; loss of balance due to dizziness; unconsciousness; taking medications for blood pressure or a heart condition; musculoskeletal, metabolic, respiratory, renal, immune, hematological, or neurological diseases; or any other reason that would prevent them from safely completing the protocol. Prior to their visit, the subjects were encouraged to consume a typical meal approximately three hours beforehand and instructed to avoid strenuous exercise and supplement ingestion for 24 h. All subjects completed a health history questionnaire, physical activity readiness questionnaire, and signed an informed consent prior to testing. The study was conducted according to the guidelines of the Declaration of Helsinki and approved by the Institutional Review Board of Northern Illinois University (IRB #HS24-0455, 20 August 2024).

### 2.2. Exercise Protocol

Subjects completed an incremental test to exhaustion on an electronically braked cycle ergometer (Lode Corival CPET, Groningen, the Netherlands). Seat height was adjusted to the greater trochanter of the subject while standing next to the cycle ergometer to ensure their legs were near full extension during each pedal revolution. Feet were secured to the pedals through foot straps to maintain pedal contact during the test. The test began at 80 watts, with the power output increasing 30 watts every two minutes until volitional fatigue or when 70 revolutions per minute could not be sustained despite strong verbal encouragement. During the test, subjects were instructed to pedal at a cadence of 70–80 revolutions per minute and strongly encouraged to continue through maximal exertion. The heart rate was recorded every 30 s through an ECG measurement and two OTBeat Burn^TM^ monitors placed on the right forearm and upper arm. The rating of perceived exertion was measured at the end of each two-minute stage using the Borg scale (6–20). The criteria for defining the maximal intensity involved the subject achieving heart rate values within 10 bpm of their age-predicted maximum and a rating of perceived exertion ≥ 18.

### 2.3. Measurement of the Heart Rate

A standard 12-lead ECG (Marquette Case Plus Stress Test Monitor, General Electric, Boston, MA, USA) with a sampling frequency of 4000 Hz was utilized as the criterion method for the heart rate. Each subject was positioned on an examination table with their shirt removed. Prior to the visit, subjects were instructed to have their chest shaved within 24 h of their scheduled time to ensure optimal skin-to-electrode contact and signal quality. The skin was lightly abraded with gauze and cleaned with isopropyl alcohol prior to placement of the electrodes (Skintact Electrodes, Leonhard Lang GmbH, Innsbruck, Austria) using the Mason–Likar configuration.

**Figure 1:**
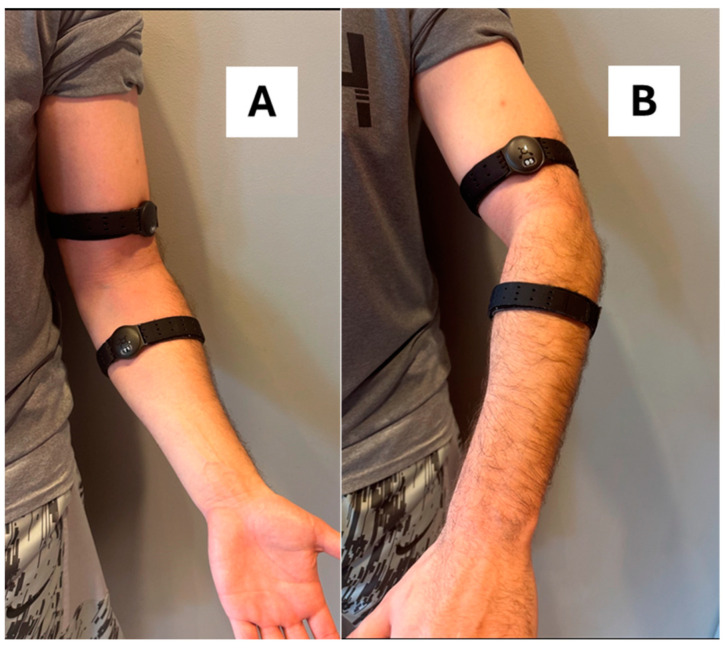
Anterior (**A**) and lateral (**B**) view of the OTBeat Burn^TM^ device location placements for the upper arm and forearm.

OTBeat Burn^TM^ heart rate monitors (Orangetheory Fitness^®^, Boca Raton, FL, USA) were placed according to the manufacturer recommendations on the right forearm and upper arm of the subject [21] (Figure 1). Specifically, the devices were tightly secured to the comfort level of the subject approximately two finger widths below the elbow for the forearm placement and two finger widths above the elbow for the upper arm placement [21] (Figure 1). The tension was standardized, so that the sensor was held firmly against the skin (without indentation or blood flow occlusion) to ensure strap movement would not occur during exercise. The monitors were then rotated in line with the strap until the sensor flashed green, indicating the heart rate was detected [21], thereby resulting in a medial placement for the forearm sensor and lateral placement for the upper arm sensor (Figure 1). Both heart rate monitors were subsequently paired via Bluetooth to separate mobile devices using the Orangetheory Mobile App and at-home indoor cycling option. The sensor lens of each device was cleaned with a soft cloth after completion of each test.

### 2.4. Data Analyses

Data were organized in Microsoft Excel (Microsoft Corp, Redmond, WA, USA) and transferred to SPSS (Version 29.0, SPSS Inc. Armonk, NY, USA: IBM Corp.) for subsequent analysis. Instantaneous heart rate values from the ECG and OTBeat Burn^TM^ monitors were recorded simultaneously every 30 s [17,18]. The four heart rate values associated with each two-minute stage were then averaged for each device. The accuracy of the OTBeat Burn^TM^ monitors was assessed through (1) calculation of the mean absolute percent error (MAPE) to determine the average error associated with each measurement, (2) simple linear regression to determine the Pearson correlation coefficient (r) and standard error of estimate (SEE), (3) Bland–Altman plots with associated calculation of the constant error (CE = mean difference for the ECG—OTBeat Burn^TM^ heart rate values), and (4) Lin’s concordance correlation coefficient (CCC) (r*_c_*) to measure the agreement for each OTBeat Burn^TM^ monitor with ECG (i.e., the degree the paired observations fall on the identity line) [8]. For the Bland–Altman plots, limits of agreement were calculated as

upper limit = (ECG heart rate − OTBeat Burn heart rate) + (SD of difference × 1.96).

lower limit = (ECG heart rate − OTBeat Burn heart rate) − (SD of difference × 1.96).

The absolute error (AE) was calculated as $\left|ECG\;heart\;rate\;-\;OTBeat\;Burn\;heart\;rate\right|$.

The absolute percent error (APE) was calculated as

$$\frac{AE}{ECG\;heart\;rate}\times 100.$$

The MAPE was calculated as the average of all APE values. Acceptable accuracy was determined using the combined criteria of Navalta et al. [12] and Jo et al. [16], which required all the following to be met: MAPE < 5%, CE < 3 bpm, SEE < 5 bpm, and Lin’s CCC ≥ 0.9. According to the recent validation recommendations of Mühlen et al. [24], the MAPE should be utilized as the primary statistical measure for determining the accuracy of wearable heart rate devices. Conservative thresholds for the MAPE and other statistical metrics were selected for practical purposes and to be consistent with similar studies [12,16,25,26,27,28,29,30] on optical heart rate sensor validation. As described by Neudorfer et al. [30], accepting a conservative MAPE of < 5% also prevents the measurement error from exceeding the heart rate ranges of intensity zones that are often used for exercise prescriptions. Simple linear regression and intraclass correlation coefficient (ICC, *R*) (two-way mixed model with absolute agreement) were utilized to assess the inter-device reliability and interpreted according to Hopkins [31]: very poor (r = 0.45 to 0.69), poor (r = 0.70 to 0.84), good (r = 0.85 to 0.94), very good (r = 0.95 to 0.994), and excellent (r ≥ 0.995). Statistical significance was set a priori at an alpha level of 0.05.

## 3. Results

**Figure 2:**
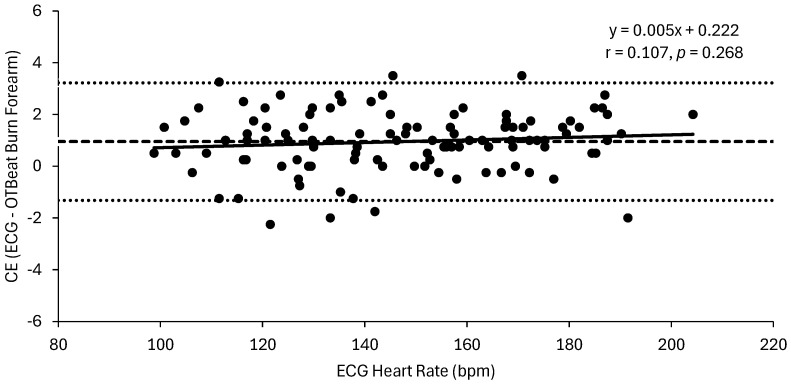
Bland-Altman plot for the relationship between the constant error (CE) (ECG-OTBeat Burn^TM^ Forearm) and the criterion ECG values. Constant error (±SD) = 1.0 (±1.2) bpm (dashed line). Upper and lower limits of agreement = 3.22 and −1.32, respectively (dotted lines). The line of regression is represented by a solid line (*n* = 109 data points).

**Figure 3:**
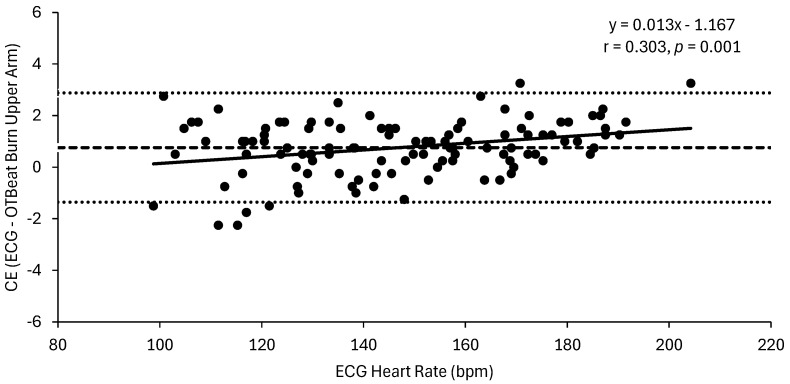
Bland-Altman plot for the relationship between the constant error (CE) (ECG-OTBeat Burn^TM^ upper arm) and the criterion ECG values. Constant error (±SD) = 0.8 (±1.1) bpm (dashed line). Upper and lower limits of agreement = 2.88 and −1.36, respectively (dotted lines). The line of regression is represented by a solid line (*n* = 109 data points).

**Figure 4:**
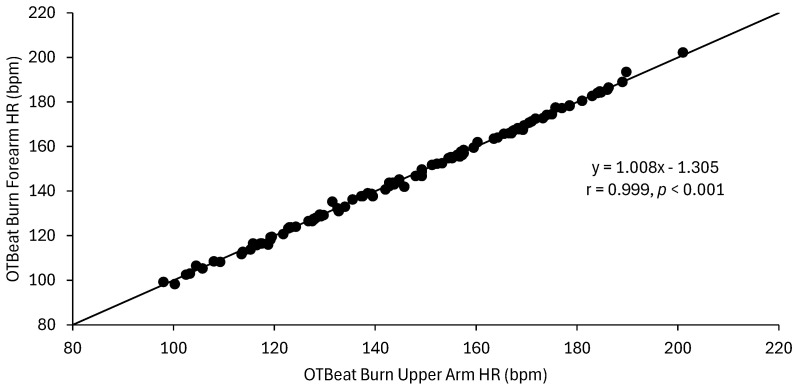
Inter-device reliability for the OTBeat Burn^TM^ heart rate monitors placed on the forearm and upper arm (*n* = 109 data points).

The MAPE (±SD) values were 0.9 (±0.6)% and 0.8 (±0.5)% for the forearm and upper arm OTBeat Burn^TM^ heart rate monitors, respectively. The linear regression analyses for the ECG vs. forearm OTBeat Burn^TM^ heart rate resulted in an excellent correlation (r = 0.998) and SEE value of 1.2 bpm, whereas the relationship for the ECG vs. upper arm OTBeat Burn^TM^ heart rate resulted in an excellent correlation (r = 0.999) and SEE value of 1.0 bpm. The mean (±SD) CE (actual ECG heart rate—predicted OTBeat Burn^TM^ heart rate) values were 1.0 (±1.2) bpm and 0.8 (±1.1) bpm for the forearm and upper arm, respectively. The Bland–Altman regression analyses (CE vs. ECG heart rate) resulted in very poor correlations for the forearm (r = 0.107, *p* = 0.268) (Figure 2) and upper arm (r = 0.303, *p* < 0.001) (Figure 3). In Figure 2, the Bland–Altman plot for the forearm placement illustrated that the CE (ECG—OTBeat Burn^TM^ heart rate) was evenly distributed across the heart rate range of exercise with upper and lower limits of agreement = 3.22 and −1.32 bpm, respectively. Thus, the heart rate values provided by the OTBeat Burn^TM^ forearm placement exhibited consistent accuracy across the exercise intensities and were at least within 4 bpm of the criterion ECG (Figure 2). The Bland–Altman plot for the upper arm (Figure 3), however, demonstrated that the CE tended to decrease at higher heart rate values, with upper and lower limits of agreement = 2.88 and −1.36 bpm, respectively. Thus, the heart rate values provided by the OTBeat Burn^TM^ upper arm placement were at least within 3 bpm of the criterion ECG (Figure 3) but tended to become more accurate at higher intensities. Lin’s CCCs were r*_c_* = 0.998 and 0.998 for the forearm and upper arm, respectively. The Pearson correlation coefficient and ICC for inter-device reliability between the forearm and upper arm OTBeat Burn^TM^ monitors were r = 0.999 and *R* = 0.999, respectively (Figure 4). Thus, there was an excellent (r = 0.999) level of agreement between the forearm and upper arm OTBeat Burn^TM^ heart rate monitors that remained consistent across the exercise intensities (Figure 4).

## 4. Discussion

This is the first study to examine the validity and inter-device reliability of the OTBeat Burn^TM^ heart rate monitor from Orangetheory^®^ Fitness. The main findings indicated the OTBeat Burn^TM^ monitor provided highly accurate and reliable heart rate values when compared to a 12-lead ECG from low to maximal exercise intensities during cycle ergometry. All the validity criteria for heart rate monitors proposed by Navalta et al. [12] and Jo et al. [16] were achieved in both locations: MAPE < 5%, CE < 3 bpm, SEE < 5 bpm, and Lin’s CCC ≥ 0.9. Specifically, both the forearm and upper arm placements of the OTBeat Burn^TM^ had acceptable MAPE (0.9 and 0.8%), SEE (1.2 and 1.0 bpm), CE (1.0 and 0.8 bpm), and Lin’s CCC (r*_c_* = 0.998 and 0.998) values, respectively. Other studies have shown a wide range of MAPE values (0.6–25.7%), depending on the specific device, mode and intensity of exercise, and location of placement on the body [6,12,13,14,15,32]. For example, Gillinov et al. [6] reported varying MAPE values for a Polar H7 chest strap (0.6–1.1%), Scosche Rhythm+ (4.8–13.1%), Apple Watch (3.2–6.5%), Fitbit Blaze (10.4–15.9%), Garmin Forerunner 235 (4.6–13.7%), and TomTom Spark Cardio (5.9–6.7%) during incremental treadmill, cycle ergometer, and elliptical (with and without arms) exercises. Of the four exercise modes, the authors [6] found that cycle ergometry resulted in the most MAPE values achieving “acceptable” criteria (i.e., <5%) in four of the six devices (Polar H7, Scosche Rhythm+, Apple Watch, and Garmin Forerunner 235). In terms of exercise intensity, conflicting findings have been reported by previous studies. At higher exercise intensities, a number of investigations have shown improved accuracy, likely due to improved blood perfusion [11,13]. Other findings [6,14,15,23,32], however, indicated reduced accuracy at higher intensities, potentially due to increased motion artifact. In the present investigation, the Bland–Altman results showed that the accuracy remained unchanged across the heart rate range (r = 0.107) for the forearm placement (Figure 2), whereas the accuracy slightly improved with the intensity at the upper arm placement (r = 0.303) (Figure 3). As proposed by Hettiarachchi et al. [11], arm-based monitors have been found to provide greater accuracy at higher exercise intensities, likely due to an increase in blood flow to the extremities. Although this may explain the improved MAPE values for endurance sports with limited arm movement (e.g., cycling and running), it has also been suggested [33] that larger limits of agreement reported during recreational activities (e.g., tennis, kayaking, and fitness) may be due to greater upper extremity movement. To this end, optimal PPG sensor accuracy requires secure contact to be maintained between the device and skin, which can be reduced with movement and associated factors such as moisture (i.e., sweating) [7]. As indicated by the consistent error values (i.e., constant accuracy) of the forearm (Figure 2) and improved error values (i.e., increased accuracy) of the upper arm (Figure 3), with an increased exercise intensity, it is likely that motion artifact did not significantly affect the OTBeat Burn^TM^ placements during cycle ergometry in the present study.

Consistent with our findings, other studies have reported highly accurate heart rate values for the Polar OH1 monitor, also placed on the forearm and upper arm, across light-to-vigorous exercise intensities on a cycle ergometer and treadmill (ICC = 0.962–0.995 [11], MAPE = 0.4% [28], and ICC > 0.998 [33]). Additional forearm and upper arm-based devices (i.e., Polar Verity Sense and Scosche Rhythm) have shown highly accurate MAPE values of <5.0% during various forms of physical activity [6,11,12,19,28,29,30,33,34,35]. For example, Scosche Rhythm monitors have exhibited acceptable MAPE values of 0.9–4.8% during cycle ergometry [6,19,30], 1.2–4.0% during treadmill exercise [19,30,35], and 3.8% during downhill trail running [12], with notable exceptions for uphill trail running (MAPE = 6.2%) [12] and elliptical exercise (MAPE = 12.4–13.1%) [6]. In addition, both Polar OH1 and Polar Verity Sense have been found to be highly accurate (MAPE < 5.0%) compared to criterion measures during self-paced trail running [29] and simulated pickleball game play [34]. Collectively, the findings of the present investigation and those of others [6,11,12,19,28,29,30,33,34,35] suggest that arm-based heart rate monitors such as the OTBeat Burn^TM^ provide acceptable accuracy from low to maximal intensities during cycle ergometry, as well as other exercise modes with higher potential for motion artifact.

The MAPE values for the OTBeat Burn^TM^ monitors of the forearm (0.9%) and upper arm (0.8%) placements were comparable to those of the Polar chest strap models (e.g., H7 and H10) (MAPE = 0.6–1.1% [6] and MAPE = 0.8% [8]), which are commonly used as field-based criterion devices [12,26,28,33,34,36]. Despite the well-documented accuracy of heart rate chest straps due to direct ECG measurement of cardiac electrical activity, arm-based monitors may potentially provide better comfort, especially with the female population, due to location and convenience on the body [11]. It is also likely that arm-based monitors are less prone to shift from their desired placement location during exercise, compared to chest strap monitors that may slip or drop towards the waist with heavy ventilation or sweating.

The available data on heart rate monitor reliability are very limited, indicating the importance of conducting more comprehensive studies on inter-device (i.e., location of placement) and intra-device (test–retest) precision. Determining strong inter-device reliability provides the user with options of device placement at different locations or body side based on personal preference and comfortability while measuring the heart rate consistently. Previous studies [25,37,38,39,40] have shown that reliability is influenced by the same factors as accuracy (mode and intensity of exercise and location of placement on the body). The current findings illustrate that the OTBeat Burn^TM^ provided excellent inter-device reliability between the forearm and upper arm locations (ICC, *R* = 0.999; Figure 4). Khushhal et al. [39] and Abt et al. [37] reported the inter-device reliability (left vs. right wrist) of the Apple Watch during incremental treadmill exercise to have ICCs of *R* = 0.91–0.99 and 0.84, respectively. In addition, the Xiaomi Mi Band 2 exhibited improved inter-device reliability (left vs. right wrist) at higher intensities, with ICC ranges of *R* = 0.47–0.71 [40], whereas the Mio Smart Bracelet provided highly variable reliability (ICCs, *R* = −0.05–0.959) based on differences in the physical activity level of the subjects and exercise intensity [25]. Although the test–retest reliability was not assessed in the present study, Climstein et al. [38] and Cai et al. [25] found intra-device ICCs of *R* = 0.58–0.92 and 0.53–0.70 during separate incremental treadmill tests to exhaustion, respectively. When compared to our findings, it can be concluded that heart rate devices designed for the wrist can influence inter-device reliability, potentially due to a relationship between motion artifact and distal placement of the device. Reliability was higher the more proximal the device placement on the body, as seen with the OTBeat Burn^TM^ having better inter-device reliability at the forearm and upper arm locations when compared to wrist-based monitors (left vs. right) [37,39]. Furthermore, the high level of reliability of the OTBeat Burn^TM^ was likely due to less motion artifact associated with cycle ergometry vs. treadmill running and potentially greater blood perfusion from gripping the ergometer handles, thereby creating a more stable environment for the assessment of the heart rate.

There were a number of limitations in the present study. First, our sample consisted of apparently healthy, college-aged males. Therefore, it is unknown if our findings can be extended to other populations due to differences that may exist in cardiovascular function (e.g., maximal heart rate, cardiac output, and endothelial function); body composition (e.g., amount of subcutaneous tissue between device and blood vessel); and other factors. Compared to females, older individuals, and clinical populations, it is likely that our sample of apparently healthy, college-aged males exhibited higher maximal heart rate, cardiac output, and blood pressures values, leading to greater blood perfusion in the forearm and upper arm that strengthened the accuracy of the OTBeat Burn^TM^ monitors with PPG sensors. Our current findings may also be limited for broader applicability in populations that exhibit larger amounts of subcutaneous fat, which may affect PPG sensitivity due to pulse signal attenuation. Moreover, Moraes et al. [22] reported that the PPG signal waveform can be significantly influenced by factors such as body age, vascular age, and level of physical activity. Thus, it is possible the current results are only applicable to healthy, college-aged males and should not be extended to other populations. Furthermore, we only examined heart rate responses during stationary cycle ergometry. Given that the OTBeat Burn^TM^ monitor is arm-based, it is possible that more dynamic exercise (e.g., running, rowing, and resistance training) could lead to greater motion artifact, thereby influencing the validity and inter-device reliability of this device. Lastly, the test–retest (i.e., intra-device) reliability was not examined in the present investigation. Future studies should prioritize multi-session trials on separate days to strengthen the overall reliability claims for a particular wearable heart rate monitor.

## 5. Conclusions

In summary, the findings of the present study indicated that the OTBeat Burn^TM^ exhibited highly acceptable validity metrics (i.e., MAPE < 5%, CE < 3 bpm, SEE < 5 bpm, and Lin’s CCC ≥ 0.9) during incremental cycle ergometry in healthy, college-aged males. Furthermore, there was a high degree of inter-device reliability (ICC, *R* = 0.999) between the forearm and upper arm placements across the heart rate range consistent with high-intensity exercise. Thus, the OTBeat Burn^TM^ is an acceptable heart rate monitor for Orangetheory^®^ Fitness members that provides accurate and reliable values and can be useful for assisting in the maintenance of heart rate zones for achieving personal goals. Future studies should examine the OTBeat Burn^TM^ monitor in other modes of exercise due to its high accuracy and precision during cycle ergometry.

## Data Availability

The raw data supporting the conclusions of this article will be made available by the corresponding author (ccamic1@niu.edu) upon request.

## Abbreviations

The following abbreviations are used in this manuscript:

MAPE	Mean absolute percent error
CCC	Lin’s concordance correlation coefficient
ECG	Electrocardiogram
ICC	Intraclass correlation coefficient
PPG	Photoplethysmography
CE	Constant error
AE	Absolute error
APE	Absolute percent error
SEE	Standard error of estimate
